# Transcription-Replication Collisions and Chromosome Fragility

**DOI:** 10.3389/fgene.2021.804547

**Published:** 2021-12-10

**Authors:** Wei Wu, Jing Na He, Mengjiao Lan, Pumin Zhang, Wai Kit Chu

**Affiliations:** ^1^ Zhejiang Provincial Key Laboratory of Pancreatic Diseases, The First Affiliated Hospital of Zhejiang University, Hangzhou, China; ^2^ Department of Ophthalmology and Visual Sciences, The Chinese University of Hong Kong, Hong Kong, Hong Kong, China

**Keywords:** transcription, replication, mitotic DNA synthesis (MiDAS), replication stress, fragile sites

## Abstract

Accurate replication of the entire genome is critical for cell division and propagation. Certain regions in the genome, such as fragile sites (common fragile sites, rare fragile sites, early replicating fragile sites), rDNA and telomeres, are intrinsically difficult to replicate, especially in the presence of replication stress caused by, for example, oncogene activation during tumor development. Therefore, these regions are particularly prone to deletions and chromosome rearrangements during tumorigenesis, rendering chromosome fragility. Although, the mechanism underlying their “difficult-to-replicate” nature and genomic instability is still not fully understood, accumulating evidence suggests transcription might be a major source of endogenous replication stress (RS) leading to chromosome fragility. Here, we provide an updated overview of how transcription affects chromosome fragility. Furthermore, we will use the well characterized common fragile sites (CFSs) as a model to discuss pathways involved in offsetting transcription-induced RS at these loci with a focus on the recently discovered atypical DNA synthesis repair pathway Mitotic DNA Synthesis (MiDAS).

## Introduction

To proliferate, a cell needs to go through a division cycle, where it duplicates its chromosomes in S phase. The replicated chromosomes are then separated and segregated into daughter cells during mitosis. Due to the large size of human genome, cells start DNA replication from multiple origins and up to thousands of replication forks are established and coordinated to replicate the genome in a very short time period (S phase). The untimely duplication of the whole genome in S phase can lead to cells entering mitosis with under replicated DNA (URD). URD can affect sister chromatids separation and genome stability, which is a hallmark of cancer (Hanahan and Weinberg, 2011).

There are certain regions in the human genome that are inherently hard-to-replicate, posing a great challenge for the timely duplication of the entire genome. Such regions including fragile sites, rDNA and telomeres have been well documented and are the major cause of chromosome fragility (Özer and Hickson, 2018). Intriguingly, many of these regions are either characterized as containing very large genes or harboring highly transcribed genes, indicating transcription might play an important role in determining their fragility. Indeed, for very long genes that take more than one cell cycle for them to be transcribed, collisions of transcription and replication machineries are inevitable (Helmrich et al., 2011). In this article, we will first review the transcription-replication conflicts and the associated DNA:RNA hybrid called R-loop. R-loop can trigger RS leading to chromosome fragility. We will then discuss the strategies employed by cells to counteract this transcription associated RS to maintain chromosome stability.

## Transcription-Mediated Replication Obstacles

During transcription, RNA polymerase (RNAP) together with multiple transcription elongation and RNA processing factors form a large complex, which is tightly bound to DNA. In addition, eukaryotic transcription needs to be coupled with other downstream events, like RNA splicing to generate mature mRNA. Thus, transcription machineries together with RNA processing factors could be obstacles for an advancing replication fork. Since DNA replication and transcription compete for the same DNA template, it is inevitable that on some occasions there will be a collision between the two machineries. Indeed, it has long been known that transcription can trigger replication-dependent genome instability that would lead to recombination and mutations (Keil and Roeder, 1984; Voelkel-Meiman et al., 1987; Chávez and Aguilera, 1997; Prado and Aguilera, 2005; Kim and Jinks-Robertson, 2012).

Depending on how transcription and replication machineries approach each other, they can either collide co-directionally or in a head-on manner. When transcription uses the leading stand of DNA replication as a template, co-directional collisions might happen; when transcription uses the lagging strand as the substrate, head-on collisions might take place. In general, it is thought that a co-directional encounter is less toxic to cells than the head-on collision. Consistent with this notion, an *in vitro* study suggested that a reconstituted *Escherichia coli* replisome can remove or bypass a co-directional RNAP and use the newly synthesized mRNA as a primer to carry on DNA synthesis (Liu et al., 1993; Pomerantz and O’Donnell, 2008). Another fact is that highly transcribed regions of rRNA, tRNA and some other essential genes are almost exclusively co-directional to fork progression in most of the studied bacteria (Rocha and Danchin, 2003; Guy and Roten, 2004). This is probably because of the evolutionary pressure for the proper replication fork progression at these regions, which is critical for genome stability and cell survival. Indeed, inverting the highly transcribed ribosomal RNA (rRNA) operons can severely affect replication fork progression at these regions and induce DNA damage responses and cell death (Srivatsan et al., 2010). Intriguingly, there is also a bias towards co-directional collision between transcription and replication in the human genome (Petryk et al., 2016). On the other hand, co-directional encounters of transcription and replication machineries can also cause DNA damages. For example, a co-directional encounter can induce replication restart with the help from helicases and replication restart proteins in *Bacillus subtilis* (Merrikh et al., 2011). In budding yeast, DNA polymerases tend to accumulate at highly transcribed genes in an orientation independent manner (Azvolinsky et al., 2009). Furthermore, by generating an episomal system in human cells, Hamperl *et al.* discovered that co-directional collisions can provoke ATM-dependent DNA damage responses (Hamperl et al., 2017).

Multiple experimental data has indicated that head-on collisions are more deleterious to fork progression than co-directional collisions and are commonly associated with DNA damage formation (Deshpande and Newlon, 1996; Prado and Aguilera, 2005; Merrikh et al., 2012). First, the tightly bound large RNAP complex can be a physical barrier that is difficult for the replisome to bypass. Second, when transcription and replication machineries move towards each other, the accumulated positive supercoiled DNA structures would slow down the progression of replication fork (Bermejo et al., 2012). Consistent with this, torsional stress reliving factors: DNA topoisomerases I and II, travel with replication forks and are required for the avoidance of transcription and replication conflicts (Tuduri et al., 2009; Bermejo et al., 2012). Lastly, head-on collisions are always associated with the formation of stable pathological nucleic acid structure: R-loop. R-loop is a three-stranded nucleic acid structure containing one DNA-RNA hybrid and one single strand of DNA. It is much more stable than double stranded DNA, and therefore can directly interfere with DNA replication leading to fork stalling or collapse (Aguilera and García-Muse, 2012; Santos-Pereira and Aguilera, 2015). Consistent with this idea, R-loops are significantly enriched at head-on regions in human cells, and introducing a head-on collision on a plasmid causes plasmid loss in a R-loop dependent manner (Hamperl et al., 2017).

## Transcription at Chromosomal Fragile Loci

As mentioned above, certain regions in the human genome are intrinsically difficult to replicate and are prone to recombination and viral integration (Thorland et al., 2003; Gao et al., 2017). These regions are called chromosomal fragile loci. Accumulating evidence suggests that transcription and replication conflict is an important factor contributing to chromosomal fragility. Some of these fragile regions are discussed below.

### Fragile Sites

Fragile sites are specific regions that tend to form gaps or breaks on metaphase chromosomes in the presence of RS (Sutherland, 1979). Fragile sites could potentially comprise genome stability and are highly associated with the development of various diseases, including cancer and neurological diseases. Currently, there are three known types of fragile sites:

Rare fragile sites (RFSs) are only found in less than 5% individuals in the general population, and their fragility is associated with the expansion of dinucleotide or trinucleotide repeats, which can form secondary DNA structures, affecting DNA replication and transcription (Magenis et al., 1970). Furthermore, R-loop has been found to be at RFS:FRAXA, and is associated with its silencing (Kumari et al., 2015; Bhowmick et al., 2016).

**FIGURE 1:**
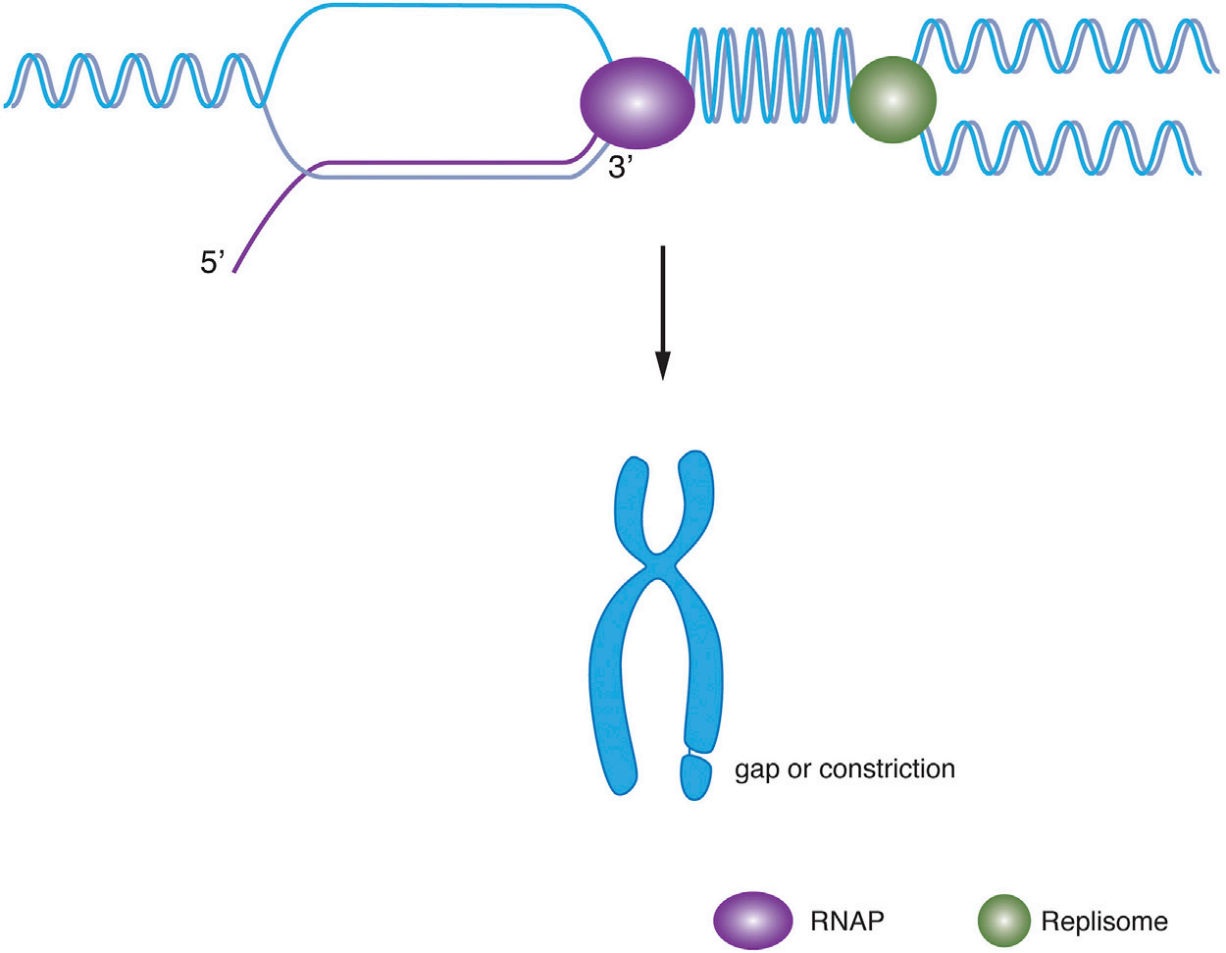
Model for head-on collision and chromosome fragility. A head-on collision of the RNA polymerase (purple, left) with replisome (green, right) results in supercoiling and R-loop leading to chromosome fragility.

Common fragile sites (CFSs) are present in all individuals. Some of the characteristics of CFSs might explain their fragility. First, CFSs are late replicating, which might leave insufficient time for cells to clear DNA replication obstacles or to repair damaged DNA following replication perturbations (Le Beau, 1998; Hellman et al., 2000). Second, CFSs are short of replication origins (Letessier et al., 2011; Sugimoto et al., 2018; Pruitt et al., 2017). Thus, to completely duplicate CFSs, two replication forks need to travel a long distance to converge, which is challenging to replication forks, especially when cells experience RS. Recent evidence suggests CFSs fragility is tightly associated with transcription. Indeed, CFSs harboring actively transcribed long genes tend to be hotspots for copy number variants (CNVs) (Wilson et al., 2015). Interestingly, more than 80% of human CFSs and all mouse embryonic flbroblast CFSs overlap with long genes of more than 300 kb (et al., 2013). Due to their large sizes, these regions can take the entire cell cycle for them to be completely transcribed, therefore the collisions between replication and transcription are inevitable, creating stable R-loops and consequent CFSs fragility (Helmrich et al., 2011) (Figure 1). Consistent with this notion, the depletion of R-loop by RNase H1 overexpression can reduce CFSs fragility (Helmrich et al., 2011). Also, low doses of aphidicolin (APH, commonly used as a CFSs fragility inducer) induced the formation of FANCD2 protein foci, which decorate the location of CFS loci, were found to bind to the central regions of large genes in human and chicken DT40 cells (Pentzold et al., 2018). In addition, FANCD2 has also been shown to be required to promote DNA replication at CFSs by preventing R-loop formation (García-Rubio et al., 2015; Schwab et al., 2015; Madireddy et al., 2016). Our recent study has identified RTEL1 as a key factor in suppressing CFSs fragility by resolving transcription-replication conflicts caused by G-quadruplex and R-loop structures. Moreover, through DNA-RNA immunoprecipitation (DRIP) sequencing, we found both RTEL1 and low dose APH can induce genome-wide R-loop formation. Importantly, around 70% of well characterized CFSs contains at least one region with high signal intensity of R-loop, which are significantly enriched following RTEL1 depletion or APH treatment (Wu et al., 2020). Therefore, transcription and replication collisions and their associated R-loops might underline CFSs fragility.

Early replicating fragile sites (ERFSs) are newly discovered fragile sites that reside in highly transcribed, early replicating genes. Besides, ERFSs are very close to replication origins, which further increases the incidence of transcription and replication collisions (Barlow et al., 2013; Mortusewicz et al., 2013). Recent data suggested that transcription and replication conflicts might explain the mechanism of oncogene-induced replication stress. It has been proposed that oncogene activation forces cells exiting G1 phase and entering S phase prematurely, where transcription is not able to suppress replication origins within the body of such genes, resulting in transcription and replication conflicts. Of note, ERFSs have been mapped as hotspots for sites of oncogene-induced replication and transcription collisions (Halazonetis et al., 2008; Macheret and Halazonetis, 2018).

### rDNA

Ribosomal DNA (rDNA) are regions clustered with long tandem repeats, where rRNA are transcribed. rDNA is highly transcribed by RNA polymerase I to meet the demand for the translation activity in cells. Each copy of a repeat harbors a potential replication origin (Linskens and Huberman, 1988). In yeast, it is estimated that each active rDNA gene can accommodate up to 150 RNA polymerases (French et al., 2003), which can form a physical barrier for the replication machinery (Berger et al., 1997; Coffman et al., 2005). Therefore, there are very high chances of transcription and replication collisions at rDNA (Deshpande and Newlon, 1996; Takeuchi et al., 2003; Kobayashi, 2014). In agreement with this, transcription and replication conflicts associated with R-loops are commonly found at rDNA (Chen et al., 2017; Lindström, 2018). Immunofluorescence staining analysis using a specific antibody recognizing R-loops showed the strongest signal in the nucleoli. Nuclear regions containing lots of rDNA, implying stable R-loops formation at rDNA loci. In addition, proteins involved in R-loops prevention or resolution, such as RNase H1, PIF1, and Top I are found to accumulate at rDNA region and are essential for their integrity (El Hage et al., 2010; El Hage et al., 2014; Shen et al., 2017; Tran et al., 2017). Moreover, the RNAi (RNA interference) component, Dicer, was found to be required to limit transcription and replication collisions at rDNA through promoting transcription termination (Castel et al., 2014).

### Telomere

Telomeres are regions composed of thousands of TTAGGG repeats that lie at the ends of chromosomes. Usually, telomeres are bound by the shelterin complex. These nucleoprotein structures can prevent telomeres from being recognized as DNA double strand breaks (DSBs), which is critical for genome stability and cell survival (Palm and de Lange, 2008; de Lange, 2009). Although, telomeres are heterochromatic, transcription still happens from the subtelomeric regions towards the end of chromosomes. Currently, two types of telomere associated RNA have been reported, telomeric repeat-containing G-rich RNA (TERRA) and the complementary C-rich transcripts (ARIA) (Kwapisz and Morillon, 2020). Although the biological functions of ARIA remain to be explored, TERRA has been shown to invade into telomeres and induce the formation of stable R-loop structures (Azzalin et al., 2007). TERRA R-loops can be found in all eukaryotes (Rippe and Luke, 2015). As mentioned above, stable R-loops can be a challenging obstacle for replication forks. Furthermore, the displaced G-rich single strand DNA during TERRA R-loop formation promotes the generation of another noncanonical secondary DNA structure: G-quadruplex (G4). G4 is also known to obstruct DNA replication resulting in RS and genome instability (Wang et al., 2019). Therefore, TERRA R-loop may be a major source for telomere fragility. Interestingly, the level of TERRA transcription is negatively correlated with DNA replication process, with the highest being at the G1-S transition and the lowest at the S-G2 transition (Flynn et al., 2015), which can reduce the chance of replication machineries colliding with TERRA R-loops. Besides, several R-loops processing factors have been found to be essential for telomere stability. Specifically, RNaseH1 can regulate TERRA-telomeric DNA hybrids to maintain telomere stability in cells employing non-telomerase approach to maintain the length of their telomers (Arora et al., 2014). FEN1 and UPF1 (Up-frameshift 1) can promote telomeric leading stand DNA synthesis by processing TERRA R-loops through the flap endonuclease activity and 5′-3′ helicase activity, respectively, (Azzalin et al., 2007; Teasley et al., 2015).

## Pathways in Dealing With Transcription Associated RS at CFSs

Despite the high incidence of transcription and replication collisions and the prevalence of R-loops at chromosome fragile loci, cells seem to proliferate with a fairly stable genome. Therefore, it is conceivable that cells have evolved surveillance pathways to avoid or minimize the deleterious effects cause by these transcription and replication collision events. Indeed, multiple strategies or pathways have been identified that can prevent or resolve transcription and replication collisions and R-loops, such as spatial and temporal separation of transcription and replication (Wei et al., 1998), the coupling of transcription with mRNA processing (Aguilera and García-Muse, 2012; Huertas and Aguilera, 2003; Li and Manley, 2005), RNAP anti-backtracking and clearance (Castel et al., 2014; Saponaro et al., 2014; Urban et al., 2016; Cheung and Cramer, 2011; Thomas et al., 1998; Roghanian et al., 2015; Ganesan et al., 2012; Epshtein et al., 2014; Zaratiegui et al., 2011; Zatreanu et al., 2019), Fanconi anemia pathway (Madireddy et al., 2016; Schwab et al., 2015; García-Rubio, 2015a), ATR-mediated DNA damage response and the expression of specific R-loop processing factors including RNase H1 (Lockhart et al., 2019) and SETX (García-Muse et al., 2019). Some of the key proteins involved in these pathways are summarized in Table 1. Interestingly, deficiency in some of these proteins such as FANCD2, ATR, RNase H1, and Dicer, have been linked to CFSs fragility (Casper et al., 2002; Madireddy et al., 2016; Di Marco et al., 2017; Fragkos et al., 2019), indicating cells employ these pathways to counteract transcription induced obstacles or RS to maintain CFS stability. Of note, most of these pathways seem to largely operate in S phase of the cell cycle.

**TABLE 1:** Key proteins involved in preventing or resolving transcription associated RS.

Pathways	Proteins
Transcription and mRNA processing coupling	THO complex (Gómez‐González, (2011), Sin3A (Salas‐Armenteros, (2017), Splicing factors (ASF/SF2) (Li and Manley, (2005)
RNAP anti-backtracking	TFIIS Zatreanu et al. (2019), RECQ5 (Saponaro et al., (2014))
RNAP clearance	TC-NER factors (XPF, XPG and CSB) (Sollier et al., (2014), Def1 (Wilson et al., (2013), Dicer (Castel et al., (2014)
Fanconi anemia pathway	FANCD2 (García-Rubio, (2015b)
ATR-mediated DNA damage responses	ATR (Hodroj et al., (2017); Matos et al., (2020)
R-loop processing factors	RNase H1 and RNase H2 (Lockhart et al., (2019), SETX Cohen et al., (2018), DXH9 Cristini et al., (2018), DDX39B Pérez-Calero, (2020)
Mitotic DNA synthesis pathway	POLD3, RAD52, SLX4, RTEL1 (Minocherhomji et al., (2015); Bhowmick et al., (2016); Wu et al., (2020)

Recently, an unscheduled mitotic DNA synthesis (MiDAS) pathway functioning in early mitosis has been found to be essential for CFS stability and is recognized as a “salvage pathway” to alleviate RS accumulated at CFSs (Minocherhomji et al., 2015). In response to RS, CFSs can remain under-replicated from interphase till mitosis without necessarily activating G2/M checkpoint. In early mitosis, under-replicated DNA (URD) can activate MiDAS to complete the under-replicated regions. Failure in filling up URD can cause DNA bridges (chromatin bridges and ultra-fine DNA bridges), which can affect sister chromatids separation and lead to genome instability or mitotic catastrophe (Ying et al., 2013). MiDAS is thought to be a break-induced replication (BIR) like pathway. Consistently, more than half of MiDAS foci, defined by visualizing nascent DNA synthesis in mitosis, were found to be on only one of the two sister chromatids, which is consistent with the conserved DNA synthesis feature in BIR (Özer and Hickson, 2018). Until now, many proteins have been identified to be essential for MiDAS. TRAIP is an E3 ubiquitin ligase required for replisome unloading at G2/M boundary, which is a prerequisite for SLX4-dependent endonucleases to access and cleave incomplete DNA structures. After that, RAD52 and POLD3 dependent DNA synthesis can take place. Our recent data has linked R-loop to MiDAS (Wu et al., 2020). In the presence of RS, RNase H1 depletion can stimulate robust MiDAS, while overexpression of RNase H1 can reduce MiDAS. The simplest and more understandable interpretation to these results could be envisaged as R-loop induced RS cannot be completely resolved in interphase, making the cells enter mitosis with incompletely replicated DNA, and consequently triggering the MiDAS pathway. However, there is another possibility that R-loop might directly take part in MiDAS pathway and regulate MiDAS. Indeed, R-loop can stimulate RAD52-POLD3-mediated BIR at ROS-induced telomeric DNA breaks (Tan et al., 2020). Which of the two possibilities is closer to what truly happens in cells warrants further investigation.

## Conclusion

Tumorigenesis is a process involving mutations in tumor suppressor genes and the activation of oncogenes. In the early stage of tumor, oncogene activation induces RS and drives genomic instability. Many oncogenes act as growth factors to support proliferation by upregulating transcription factors which in turn can stimulate RNA synthesis (Kotsantis et al., 2016). The increased level of transcription might enhance the conflicts between transcription and replication machineries and its associated R-loops, giving rise to transcription associated RS in S phase. Indeed, accumulating evidence suggests transcription mediated RS is one of the major sources of genome instability, especially at difficult-to-replicate loci mentioned above. Of note, these loci are preferably targeted by RS generated during cancer development (Tsantoulis et al., 2008; Barlow et al., 2013), indicating these loci might be constantly being challenged and mutated during tumorigenesis. Understanding the mechanisms underlying their fragility and identifying molecular pathways in preventing or resolving transcription associated RS will help us understand the molecular basis of cancer development and identify more cancer specific druggable targets.
